# Increasing expression of hypoxia-inducible proteins in the Barrett's metaplasia–dysplasia–adenocarcinoma sequence

**DOI:** 10.1038/sj.bjc.6603744

**Published:** 2007-04-17

**Authors:** E A Griffiths, S A Pritchard, S M McGrath, H R Valentine, P M Price, I M Welch, C M L West

**Affiliations:** 1 Academic Radiation Oncology, Division of Cancer Studies, Christie Hospital, The University of Manchester, Wilmslow Road, Withington, Manchester M20 4BX, UK; 2 Department of Gastrointestinal Surgery, University Hospitals NHS Trust, South Manchester, Manchester M23 9LT, UK; 3 Department of Histopathology, University Hospitals NHS Trust, South Manchester, Manchester M23 9LT, UK

**Keywords:** Barrett's oesophagus, HIF-1*α*, HIF-2*α*, VEGF, hypoxia

## Abstract

Hypoxia-associated markers are involved in the progression of several malignancies, but are relatively unstudied in Barrett's carcinogenesis. Our aim was to assess the immunohistochemical expression of hypoxia-inducible factor (HIF)-1*α*, HIF-2*α*, erythropoietin (Epo), Epo receptor (Epo-R), Glut-1 and vascular endothelial growth factor (VEGF) along with Ki67/MIB-1 in the Barrett's metaplasia–dysplasia–adenocarcinoma sequence. Endoscopic biopsies of normal squamous epithelium (NSE) (*n*=20), columnar-lined oesophagus (CLO) (*n*=15), CLO with intestinal metaplasia (*n*=20), dysplasia (*n*=17) and Barrett's type adenocarcinoma (*n*=20) were obtained. Immunohistochemistry was performed on the paraffin-embedded tissue. A score was calculated for each marker (range 0−300) by multiplying intensity (none 0, weak 1, moderate 2, strong 3) by percentage of expression (range 0–100). Significant increases in the expression of HIF-2*α* (*P*=0.014), VEGF (*P*<0.0001), Epo-R (*P*<0.0001) and Ki67 (*P*<0.0001) were found as tissue progressed from NSE to adenocarcinoma. HIF-2*α* was expressed late in the sequence and was only seen in dysplasia and adenocarcinoma. High HIF-2*α* expression was seen in 12 out of 20 Barrett's type adenocarcinoma. The late expression of HIF-2*α* in the Barrett's carcinogenesis sequence and its high expression in adenocarcinoma suggest that it is worth further investigation as a marker of disease progression and therapeutic target.

Barrett's oesophagus is a pre-malignant condition associated with a 30−125-fold increase in the risk of developing adenocarcinoma compared with unaffected individuals (Guindi and Riddell, 2003). The normal squamous oesophageal lining is transformed via injury caused by gastro-oesophageal reflux disease into columnar-lined epithelium. The subsequent development of specialised intestinal metaplasia (IM) is a key risk factor for tumorigenesis (Spechler and Goyal, 1996). Additional genetic and epigenetic events are associated with further progression through increasingly severe degrees of dysplasia to adenocarcinoma. This process is termed the Barrett's metaplasia–dysplasia–adenocarcinoma sequence (Jankowski *et al*, 1999). Oesophageal adenocarcinoma is a devastating disease, which is increasing in incidence. In Europe, only about a quarter of all oesophageal cancers are operable and, for these, 5-year survival is around 20−30% (Keighley, 2003). Regular endoscopic surveillance is recommended for patients with Barrett's metaplasia because of their elevated risk of developing adenocarcinoma (Bergman and Tytgat, 2005). Cancers detected in such programmes are frequently early stage and have a better prognosis (Streitz *et al*, 1993; Corley *et al*, 2002). However, these analyses are subject to confounding factors such as lead and length time bias, and it remains to be established conclusively if surveillance is beneficial.

The exact molecular mechanisms of Barrett's adenocarcinoma development remain largely unknown, but there is interest in investigating molecular biomarkers for predicting patient risk of disease progression (Bergman and Tytgat, 2005). The rationale underlying such work is to find approaches for identifying high-risk patients who would be targeted for surveillance endoscopy (Preston and Jankowski, 2006). Although many potential biomarkers have been assessed, none is currently in routine clinical use (McManus *et al*, 2004).

Hypoxia is implicated in the progression of several malignancies, but is relatively unstudied in the Barrett's carcinogenic sequence. Tumour hypoxia is a key factor driving the development of malignancy, and the master regulatory protein that controls the response of cells to changing oxygen levels is hypoxia-inducible factor-1 (HIF-1). HIF-1 consists of constitutively expressed HIF-1β complexed with one of three subunits (HIF-1*α*, HIF-2*α* or HIF-3*α*). In hypoxia, HIF-1 upregulates genes involved in a variety of cellular processes that include glucose metabolism, erythropoiesis, cell proliferation, angiogenesis and apoptosis. Studies have shown progressively increased HIF-1*α* expression in breast (Bos *et al*, 2001), skin (Costa *et al*, 2001), gastric (Griffiths *et al*, 2007) and cervical (Acs *et al*, 2003) cancer development. Hypoxia-inducible factor-1*α* is a key mediator of angiogenesis via activation of vascular endothelial growth factor (VEGF) (Forsythe *et al*, 1996), which is associated with oesophageal carcinogenesis (Couvelard *et al*, 2000; Auvinen *et al*, 2002; Lord *et al*, 2003; Mobius *et al*, 2003). Hypoxia-inducible erythropoietin (Epo) regulates erythropoiesis by stimulating the growth and differentiation of red blood cell precursors (Yasuda *et al*, 2003). Erythropoietin and erythropoietin receptor (Epo-R) are expressed in a number of cancers and are involved in breast (Acs *et al*, 2002), endometrial (Acs *et al*, 2004), melanoma (Kumar *et al*, 2005) and prostate (Feldman *et al*, 2006) tumorigenesis. The following study was established to investigate the hypothesis that hypoxia plays a role in the aetiology of oesophageal cancer. The specific goals of the research were to determine whether the expression of hypoxia-associated proteins increases along the Barrett's carcinogenic sequence and to highlight potential markers of interest for further study as predictors of disease progression in patients with Barrett's dysplasia. Four hypoxia-associated markers were selected that have not been assessed in the Barrett's sequence: HIF-1*α*, HIF-2*α*, Epo, Epo-R. Although studied previously, VEGF and Glut-1 were studied also. The widely investigated Ki67 was included as a comparator. The expression of the proteins was assessed using immunohistochemistry in paraffin-embedded material representing the Barrett's metaplasia–dysplasia–adenocarcinoma sequence.

## METHODS

### Endoscopic biopsies

The study was approved by the South Manchester Ethics Committee. Paraffin-embedded endoscopic biopsies of 20 normal squamous epithelium (NSE), 15 columnar-lined oesophagus (CLO), 20 CLO with IM, 17 dysplasia and 20 Barrett's type adenocarcinoma were obtained from the pathology archive of South Manchester University Hospitals NHS Trust. Histopathology and endoscopy reports were reviewed to confirm biopsy location in the oesophagus.

Haematoxylin and eosin slides were reassessed by a consultant upper gastrointestinal pathologist (SAP) to select the most suitable specimens for immunohistochemistry. Biopsy samples were classified using internationally agreed criteria (Ibrahim, 2000; Schlemper *et al*, 2000; Odze, 2006). NSE was defined as normal squamous epithelium with no evidence of adjacent CLO or IM. Seventeen of the biopsies designated NSE had additional fragments of adjacent normal gastric cardia mucosa (NCM) used as additional controls to explore changes due to metaplastic transformation. CLO was defined as the presence of columnar metaplasia of the lower oesophagus without evidence of IM. CLO with IM was defined as columnar metaplasia of the lower oesophagus associated with specialised IM (characterised by goblet cells). Dysplasia was defined as unequivocal neoplastic epithelium strictly confined within the basement membrane of the gland from which it arises. This was classified as low (*n*=10) and high (*n*=7) grade depending on the degree of abnormality present. Adenocarcinoma was defined as invasive malignancy of adenocarcinoma cell type arising from the lower oesophagus in association with CLO.

### Immunohistochemistry

As deterioration can occur in stored sections (Bertheau *et al*, 1998; Olapade-Olaopa *et al*, 2001), staining was carried out within 3 months of cutting. Sections (4 *μ*m) were dewaxed in xylene and rehydrated using a series of ethanol solutions of increasing dilution.

### HIF-1*α* immunohistochemistry

Sections were microwaved for 25 min in 10 mM sodium citrate buffer solution (pH 6.0) and endogenous peroxidase quenched with 0.03% hydrogen peroxide in methanol. HIF-1*α* was detected using the Tyramide Signal Amplification System (PerkinElmer Life Sciences, NEL 700A001KT Beaconsfield, UK), which is based on streptavidin–biotin–horseradish peroxidase complex formation. Following the blocking step described in the manufacturer's protocol, the primary antibody was applied (Table 1). Biotinylated rabbit anti-mouse (DakoCytomation, E0413, Ely, UK) diluted 1 : 400 was used as the secondary antibody and the sections were incubated for 30 min at room temperature. Additional blocking precautions were employed at this stage in order to minimise the amplification of nonspecific background (Kim *et al*, 2003). The antibody was visualised using diaminobenzidine (DakoCytomation, UK) and sections counterstained with haematoxylin, dehydrated and mounted.

### HIF-2*α*, Epo, Epo-R, VEGF, Glut-1 and Ki67 immunohistochemistry

Antigen retrieval was carried out where necessary by microwaving for 25 min in either 10 mM sodium citrate (pH 6.0) or 0.05 M Tris-HCl/1 mM EDTA (pH 8.5 or 9.0) buffer solution (Table 1). After quenching endogenous peroxidase, nonspecific binding was blocked using 10% casein (Vector Laboratories, SP-5020, Peterborough, UK). The primary antibody was applied and the sections were incubated as described (Table 1). Mouse or rabbit (EnVisionPlus System; DakoCytomation, UK) secondary antibodies were used to detect the antigen using a 30-min incubation at room temperature. Following visualisation with diaminobenzidine, sections were counterstained with haematoxylin, dehydrated and mounted. In each case, substitution of the primary antibody with an identical concentration of immunoglobulins (IgG1; DakoCytomation, UK) from the same species served as a negative control. Positive and negative (or low) tissue controls from gastric, cervical or head and neck cancer with known staining characteristics were used in each batch. Batch-to-batch variation was assessed by choosing two sections showing high and low protein expression and running additional sections from these biopsies with each batch.

### Immunohistochemical scoring method

A score was calculated for each marker (range 0–300) by multiplying intensity (none 0, weak 1, moderate 2, strong 3) by percentage of expression (range 0–100). Scoring was performed blind by two independent pathologists (SAP, SMG). Any disagreement was resolved by discussion to obtain a final score.

### Statistics

Spearman's rank non-parametric test was used to calculate interobserver and intermarker correlations. To assess possible differences in expression levels in the carcinogenesis sequence, the rank–sum tests Mann–Whitney (for comparison between two groups) and Kruskal–Wallis (for comparison between all biopsy groups) were applied. The non-parametric Jonckheere—Terpstra (JT) test was also used to identify ordered differences among the biopsy categories in the oesophageal carcinogenesis sequence. With this test, the null hypothesis is that the distribution does not differ across ordered categories. To test marker progression in the Barrett's carcinogenesis sequence, the categories NSE, CLO, IM, Dys and Adeno were used. All statistical tests were two-sided at the 0.05 significance level. As adjusting statistical significance depending on the number of tests performed can create problems (Perneger, 1998), no allowance was made for multiple testing.

## RESULTS

The 92 specimens were from 64 men and 29 women who had a median age of 64 (range 26–87) years. There was insufficient tissue left for scoring of between two and four sections for each marker (Table 1). Interobserver agreement for all the markers studied was highly statistically significantly correlated (*P*<0.0001 for all). The final consensus scores in terms of percentiles for each marker are shown in Table 1. There were weak, statistically significant correlations between the expression levels of a number of markers: Ki67 expression correlated with that of HIF-2*α* (*ρ*=0.38, *P*<0.001), VEGF (*ρ*=0.66, *P*<0.001) and Epo-R (*ρ*=0.67, *P*<0.001); Glut-1 expression correlated with that of HIF-1*α* (*ρ*=0.38, *P*<0.001), HIF-2*α* (*ρ*=0.38, *P*=0.003) and Epo (*ρ*=0.62, *P*<0.001); HIF-2*α* expression correlated with that of HIF-1*α* (*ρ*=0.40, *P*<0.001) and VEGF (*ρ*=0.30, *P*=0.004); Epo-R expression correlated with that of VEGF (*ρ*=0.71, *P*<0.001) and inversely with that of Epo (*ρ*=−0.28, *P*=0.009).

### Descriptive pathology

**Figure 1:**
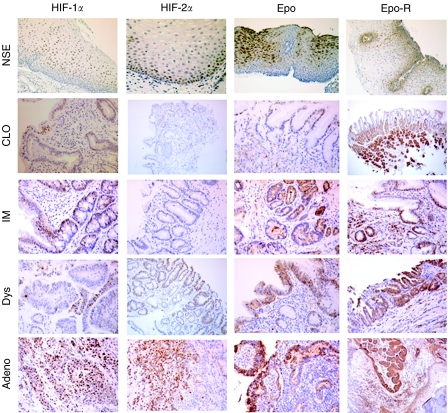
Representative photomicrographs of HIF-1*α*, HIF-2*α*, Epo and Epo-R in Barrett's metaplasia–dysplasia–adenocarcinoma sequence. NSE=normal squamous epithelium; CLO=columnar-lined oesophagus; IM, intestinal metaplasia; Dys=dysplasia; Adeno=adenocarcinoma.

Photomicrographs of the immunohistochemical expression of each marker studied are shown in Figure 1. Hypoxia-inducible factor-1*α* staining was predominantly nuclear, and occasional cytoplasmic staining was not scored. Staining was focal and tended to be intense in areas adjacent to inflammation and ulceration. Similarly, HIF-2*α* staining was predominantly nuclear and occasional focal cytoplasmic staining was ignored. Hypoxia-inducible factor-2*α* was not seen in either CLO or IM, but its expression tended to be high (>50% positive cells) in dysplasia and carcinoma. No particular preferred location of staining was identified.

VEGF was expressed in the cytoplasm. Where squamous epithelium was seen there was staining of the basal layers. In CLO and IM, staining was seen in mucous cells (fovelar-type), on the surface, in all layers of the foveolar pit and in goblet cells. In dysplasia and carcinoma, staining was random with no specific architectural pattern identified.

Erythropoietin expression was granular and cytoplasmic. Staining in CLO and IM was mainly in mucous cells located in the basal region of the cell cytoplasm surrounding the nucleus. Mucous cells in all locations stained (surface and all levels of the foveolar pit) but with a tendency for a higher percentage of surface cells to be positive. Focal staining of chief and parietal cells was also identified. Dysplasia and carcinoma had a random staining pattern. Epo-R staining was cytoplasmic with an increase in intensity around the nucleus. Lymphocytes within the lamina propria were positive for the antibody and acted as an internal control. In CLO and IM, staining was seen in mucous-secreting cells predominantly of the neck region of the foveolar pits. In these cells, the cytoplasmic staining was mainly towards the basal region of the cell around the basally placed nucleus. Staining of goblet cells was also present. In Dys and Adeno, staining was diffuse with a random location of positive cells.

Glut-1 was expressed in lymphocytes within the lamina propria, which acted as an internal control. Staining was cytoplasmic with only a few positive cases detected. No specific location of staining was identified.

Ki67 staining was nuclear. In CLO and IM, mucous cells of the deep and middle third of the foveolar pit showed positive staining with the surface region being negative. Positive staining was also seen in the glands deep to the foveolar pits. In dysplasia and carcinoma, staining was increased and present in all layers of the mucosa with loss of the normal proliferation pattern.

In some biopsies, specialised gastric body mucosa containing chief and parietal cells was present (17 NSE, 8 CLO, 3 IM, 1 dysplasia and 2 adenocarcinoma). The parietal and chief cells all showed strong staining for Epo, Epo-R and VEGF immunohistochemistry, regardless of the morphology of adjacent mucosa. This staining was not observed with HIF-1*α*, HIF-2*α*, Glut-1 and Ki67 staining. It is unclear whether this was secondary to cross-reactivity or true positive staining. Data from these areas were therefore not included in the calculated score for each biopsy.

### Marker expression along the Barrett's metaplasia–dysplasia–adenocarcinoma sequence

**Figure 2:**
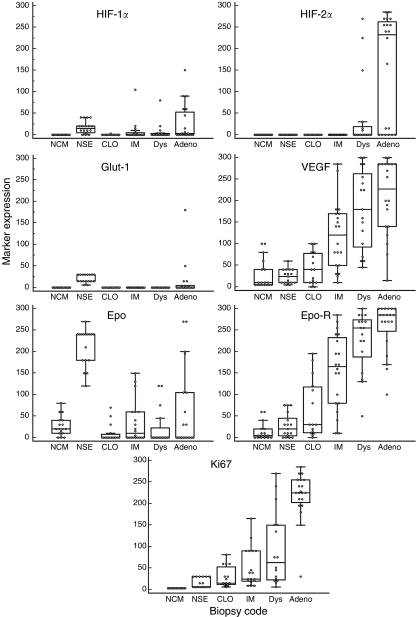
Box and whisker plots of each immunohistochemical marker in the Barrett's sequence. The box represents the 25–75 quartile with a median line. The whiskers extend to minimum and maximum values, but exclude outlying and far out values. Individual data points are also shown. NSE=normal squamous epithelium; NCM=normal gastric cardia mucosa; CLO=columnar-lined oesophagus; IM=intestinal metaplasia; Dys=dysplasia; Adeno=adenocarcinoma.

Box and whisker plots of marker expression are shown in Figure 2. Using Kruskall–Wallis testing, all markers showed significant differences in expression in the Barrett's metaplasia–dysplasia–adenocarcinoma sequence (*P*<0.001 for all). The JT test assesses ordered differences and showed significant increases in expression for HIF-2*α* (*P*=0.014), VEGF (*P*<0.0001), Epo-R (*P*<0.0001) and Ki67 (*P*<0.0001) from normal squamous tissue to adenocarcinoma. Erythropoietin, Glut-1 and HIF-1*α* showed a decrease in expression from NSE to CLO (*P*<0.001 for all). There were significant differences in marker expression in adenocarcinoma compared with dysplasia for HIF-1*α* (*P*=0.014), HIF-2*α* (*P*=0.012), Ki67 (*P*=0.0001) and Epo-R (*P*=0.042).

**Figure 3:**
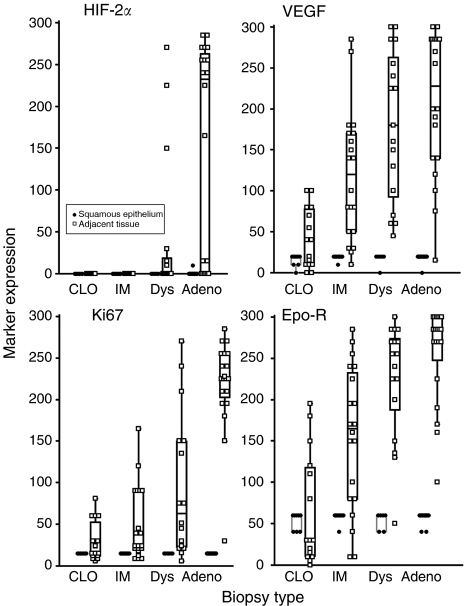
Comparison of the expression of HIF-2*α*, VEGF, Ki67 and Epo-R in normal squamous epithelium compared with adjacent columnar-lined oesophagus (CLO), intestinal metaplasia (IM), dysplasia (Dys) and adenocarcinoma (Adeno). A total of 40 biopsies were studied.

In some samples of CLO (*n*=8), IM (*n*=10), dysplasia (*n*=6) and adenocarcinoma (*n*=16), adjacent NSE was available for direct comparison. Increases in the expression of HIF-2*α*, VEGF, Ki67 and Epo-R in the relevant biopsy category compared with adjacent NSE are shown (Figure 3).

## DISCUSSION

Significant increases in the expression of HIF-2*α*, VEGF, Epo-R and Ki67 (*P*<0.0001) were found along the Barrett's metaplasia–dysplasia–adenocarcinoma sequence. Previous studies have shown the importance of Ki67 expression and proliferation in the progression of Barrett's oesophagus (Iftikhar *et al*, 1992; Reid *et al*, 1993; Polkowski *et al*, 1995; Feith *et al*, 2004). Similarly, the role of VEGF and angiogenesis in the Barrett's sequence has been widely reported and discussed (Couvelard *et al*, 2000; Auvinen *et al*, 2002; Lord *et al*, 2003; Mobius *et al*, 2003).

Our finding that Glut-1 is expressed in adenocarcinoma but not in dysplasia agrees with published work (Younes *et al*, 1997, 2000). These papers reported higher (45–69%) expression of Glut-1 in Barrett's adenocarcinoma than the 25% found here. As there was no expression in high-grade dysplasia, the authors concluded that ‘Glut-1 immunostaining may provide a unique marker that could distinguish between high grade dysplasia and a well-differentiated carcinoma, when such distinction cannot be made on purely morphological grounds’ (Younes *et al*, 1997). Our confirmatory observation adds weight to this conclusion. We also found a low percentage of Glut-1 staining in the basal layers of normal squamous oesophageal tissue, which has not previously been reported by others.

HIF-1*α* expression decreased from NSE to CLO and then increased with progression to adenocarcinoma. Although HIF-1*α* is of interest as a marker of hypoxia, its increase in expression from CLO to adenocarcinoma might be inflammatory mediated. Chronic inflammation and reactive oxygen species (ROS)-induced DNA damage are important in oesophageal tumour carcinogenesis (Olyaee *et al*, 1995; Jimenez *et al*, 2005). Reactive oxygen species regulate HIF stability and transcriptional activity under hypoxia *and* normoxia (Pouyssegur and Mechta-Grigoriou, 2006). Inflammation-mediated COX-2 expression is an early feature of Barrett's carcinogenesis (Morris *et al*, 2001) and, in a gastric cancer model, the COX-2/PGE2/HIF-1/VEGF pathway was shown to contribute to tumour angiogenesis (Huang *et al*, 2005).

As ROS are also involved in the stabilisation of HIF-2*α* (Guzy *et al*, 2005), ROS due to chronic inflammation might also play a role in the increasing expression of HIF-2 in the Barrett's sequence. The high expression of HIF-2*α* in dysplasia and adenocarcinoma makes it of interest for further investigation in a case–control study as a potential predictor of progression to malignancy in patients with dysplasia. It might have a future role in differentiating nonspecific (reactive) features that can mimic dysplasia from true dysplasia or early well-differentiated adenocarcinoma.

Epo was expressed in normal squamous oesophageal tissue and there was no increase in expression along the progression sequence. Erythropoietin receptor, however, was expressed more abundantly and staining increased from normal squamous tissue to invasive oesophageal cancer. Of interest, both were expressed earlier in the Barrett's sequence than either HIF-1*α* or HIF-2*α*. Non-hypoxia stimulation of Epo/Epo-R involves a variety of factors, including various hormones and cytokines (Hardee *et al*, 2006). In addition to regulation of red blood cell production, the pleiotropic effects of Epo/Epo-R signalling include stimulation of proliferation and angiogenesis (Hardee *et al*, 2006). Erythropoietin has been shown to act in the protection of tissue from injury and can decrease inflammation (Grasso *et al*, 2004). The early expression of Epo and its receptor in the Barrett's sequence is probably, therefore, related to their role in protection from inflammation rather than hypoxia response.

There was a reasonably high correlation between the scores of many of the immunohistochemical markers studied. This is probably a reflection of the biological similarity of the proteins studied in terms of their response to inflammation and involvement in the stimulation of angiogenesis and proliferation.

There is interest in using molecular markers to identify patients with a high risk of developing adenocarcinoma in whom surveillance endoscopy can be targeted. It is probably unlikely that a single marker will be sufficient, as there appears to be no simple evolution of genetic or molecular alterations in disease progression (Jenkins *et al*, 2002). Rather there appears to be a heterogeneous accumulation of genetic and molecular changes in the Barrett's metaplasia–dysplasia–adenocarcinoma sequence (Jenkins *et al*, 2002). As the molecular changes are known to be extremely complex, this is impeding further research. In addition, this heterogeneity may ultimately be responsible for the poor response of these tumours to therapy. Given the multiple genetic alterations, which are implicated in the natural history of oesophageal adenocarcinoma, a combination of clinical risk factors and carefully validated biomarkers might improve further the predictive value of the molecular approach (Bani-Hani *et al*, 2000; Bani-Hani *et al*, 2005) and allow targeted surveillance.

HIF-2*α*, VEGF, Epo-R and Ki67 showed increased expression with progression along the Barrett's carcinogenesis sequence. The late expression of HIF-1*α* and HIF-2*α*, key mediators of gene induction in response to hypoxia, suggests that hypoxia might not be a key factor driving oesophageal cancer development. As discussed above, ROS and inflammation are likely to be involved in the stimulation of many of the proteins studied. The conclusion from this work, therefore, is that hypoxia is probably not important in the aetiology of oesophageal cancer. The late expression of HIF-2*α* in the Barrett's sequence and its high expression in adenocarcinoma suggest that it is worth further investigation as a marker of disease progression and therapeutic target.

## Figures and Tables

**Table 1:** Summary of the immunohistochemical methods used, number of biopsies examined and overall percentiles for each immunohistochemical marker studied

**Antigen**	**Isotype**	**Source**	**Ref**	**Conc. (μg/ml^−1^)**	**Incubation**	**pH^a^**	***n***	**IHC Score^b^**
HIF-1*α*	Mouse IgG1	BD Biosciences	610958	2.5	o/n 4°C	6.0	90^c^	1.3 (0,10)
HIF-2*α*	Mouse IgG1	Cancer Research UK	E190b	6	o/n 4°C	6.0	89^c^	0 (0,0.5)
VEGF	Rabbit polyclonal	Santa Cruz Biotechnology	A-20	2	1 h 25°C	8.5	89^c^	80 (30, 192)
EPO	Mouse IgG1	R&D Systems	9C21D11	30	o/n 4°C	6.0	88^c^	7.5 (0,125)
EPO/R	Rabbit polyclonal	Santa Cruz Biotechnology	C-20	0.4	o/n 4°C	6.0	88^c^	160 (30,270)
Glut-1	Rabbit polyclonal	Alpha Diagnostics Int	GT 12-A	10	1 h 37°C	n/a	89^c^	0 (0,5)
Ki-67	Mouse IgG1	Dako-Cytomation	MIB-1	0.8	o/n 4°C	9.0	89^c^	30 (15,154)

IHC=immunohistochemistry; o/n=overnight; n/a=not applicable; conc=concentration.

a Antigen retrieval pH.

b Immunohistochemical score was calculated from percentage (0–100) multiplied by intensity (0–3) of expression for each marker studied – 50th percentile (25 and 75th percentiles).

c Insufficient tissue available for scoring in some biopsies.
